# Enhancing Medical Students’ Confidence and Knowledge in Antibiotic Prescription and Administration through Virtual Education: A Quasi-Experimental Study

**DOI:** 10.3390/antibiotics12101546

**Published:** 2023-10-16

**Authors:** Israa Abdullah Malli, Mohamud Salaad Mohamud, Sami Al-Nasser

**Affiliations:** 1 College of Medicine, King Saud Bin Abdulaziz University for Health Sciences, Jeddah 22384, Saudi Arabia; 2 King Abdullah International Medical Research Center, Jeddah 22384, Saudi Arabia; 3 Department of Primary Care and Public Health, School of Public Health, Faculty of Medicine, Imperial College London, London W6 8RP, UK; 4 Research and Development, Somali Centers for Public Health, London NW2 1TB, UK; 5 College of Medicine, King Saud Bin Abdulaziz University for Health Sciences, Riyadh 11481, Saudi Arabia; 6 King Abdullah International Medical Research Center, Riyadh 11481, Saudi Arabia

**Keywords:** antibiotic, stewardship, virtual, education, confidence, knowledge, course

## Abstract

Background: Antibiotic resistance is a worldwide health concern that necessitates antibiotic stewardship. Medical students significantly impact future healthcare practices; thus, their trust in antibiotic prescription and administration is crucial. This research aims to assess medical students’ levels of confidence and knowledge in these areas before and after exposure to virtual antibiotic stewardship education. Methods: A one-group pretest-posttest design was conducted with medical students from King Saud bin Abdulaziz University for Health Sciences in Jeddah, Saudi Arabia. Participants were enrolled in the WHO-online antibiotic stewardship course. Results: The group’s baseline confidence and background knowledge were reported to be lower than what was offered after introducing the virtual course. The McNemar–Bowker test showed a significant difference in students’ confidence in pre-course and post-course scores (Z = 20, *p* < 0.002); the matched paired *t*-test revealed a significant difference in students’ knowledge scores (M = 7.66 verses M = 5.36, Z = 3.54, *p* = 0.001). In the sample, 70% of the students were unfamiliar with antibiotic stewardship; thus, the analysis revealed a significant difference in their familiarity before and after enrolling in the online course (30 vs. 100, *p* < 0.001). Conclusion: Medical students experience low confidence in the safe practice of the antibiotic stewardship program. The WHO-online antibiotic stewardship course is considered a valuable resource that can be used in a formative medical curriculum. Thus, educators and the academic curriculum must promote practical strategies to minimize antibiotic stewardship literacy and increase antibiotic prescribing and administration quality. Introducing antibiotic stewardship across the medical curriculum and establishing educational courses are some strategies that can be undertaken to ensure that future doctors are well-educated in the principles and practices of the appropriate use of antibiotic stewardship.

## 1. Introduction

Throughout history, substantial advances in managing infectious diseases have occurred, with several discoveries and improvements emerging in recent years [1]. These improvements have aided in preventing, diagnosing, treating, and controlling infectious illnesses [2,3]. Antibiotics, antiviral medications, diagnostic tools, and vaccination are some of the significant advancements in managing infectious illnesses. However, several infectious illnesses caused by bacterial agents have resurfaced in the population [4,5]. Some serious infections are classified among the top ten leading causes of human mortality in the twentieth century [6,7]. Researchers contend that people continue to die from bacterial infections despite the availability of various classes and kinds of antibiotics due to antibiotic resistance [8,9].

Antibiotic resistance is a huge worldwide health concern that occurs when bacteria acquire the capacity to resist antibiotic actions, leaving these medications less effective or completely useless in treating diseases [10]. It has important public health consequences since it may lead to longer and more severe diseases, higher healthcare expenses, and even deaths [11]. The global burden of antibiotic resistance motivates healthcare providers and lawmakers to tackle the menace of multidrug-resistant bacteria [12,13] aggressively. Antibiotic-related malpractice and bacteria resistance are connected mainly to poor stewardship by physicians, pharmacists, and, in some cases, consumers [14]. Many studies have also shown a significant rate of incorrect antibiotic treatment when prescribing and employing medical practitioners with a wide variety of competence and experience, such as medical and dental clinicians, nurses, midwives, pharmacists, and orthodontic prescribers [15,16].

Antibiotic stewardship is an educational program that encourages, educates, and supports safe antibiotic prescription, use, and control [17]. The ultimate purpose of this program is to increase healthcare practitioners’ understanding of antibiotics to reduce antibiotic resistance, improve patient outcomes, and restrict the spread of communicable illnesses caused by multidrug-resistant organisms [18]. Health professional schools’ curricula should include adequate knowledge of antibiotic resistance guidelines to eliminate the hardships of healthcare providers with inappropriate antibiotic prescriptions in clinical practice [15]. Moreover, after university education and training, healthcare providers should be encouraged to enroll in up-to-date courses to improve their antibiotic prescription instructions, perception, understanding, and practice toward antibiotic guidelines [19,20]. These courses will expose the learners and give them enough knowledge and ability to prescribe incorrect antibiotics [16].

Several studies have examined the impact of educational courses on medical students’ knowledge and confidence in antimicrobial stewardship and antibiotic prescribing practices [21]. El-Sokkary et al. researched how an antimicrobial stewardship elective affected medical students’ knowledge and attitudes about the appropriate and effective use of antibiotics [22]. The results demonstrated that the medical students who participated greatly increased their knowledge and understanding of the various concepts related to antibiotic stewardship [22]. Additionally, Hartman and colleagues found that developing a complete antibiotic stewardship program lowered the number of antibiotic prescriptions for vulnerable elderly patients suspected of having urinary tract infections [23]. According to the findings of another study [24], an education program on antimicrobial stewardship improved the appropriateness of antibiotic prescriptions.

The World Health Organization (WHO) launched an online antibiotic stewardship course to address the urgent need to control antibiotic resistance through proper surveillance and effective prevention systems [25]. It was designed to improve the knowledge and confidence of those administering antibiotic treatments, such as researchers, doctors, nurses, pharmacists, and public health officers [19,25]. It also uses a competency-based approach that covers the fundamental principles of bacteriology, including body systems and antibiotic interactions, mechanisms of bacterial evasion of the host immune system and the development of resistance, antibiotic classes and modes of action, antibiotic susceptibility, and appropriate methods for determining the proper antibiotic [26,27]. As a result, learners should grasp the critical efficiencies of antibiotic stewardship and how to apply them in routine clinical procedures by the conclusion of the course, which should take around eight hours to complete [28].

It has been alleged that medical students lack specialized expertise and ability in the antibiotic stewardship program [29]. Furthermore, several courses concentrate on antibiotic stewardship programs given similarly by other schools to combat antibiotic misuse [30]. Despite existing antibiotic resistance control measures and antibiotic stewardship educational programs at medical schools worldwide, a recent survey of medical students in their final semester revealed low confidence in their knowledge of antibiotic options, the proper prescription, and dosing treatment [20,31]. No published survey to our knowledge assesses Saudi Arabian medical students’ current understanding of antibiotic stewardship that they have obtained throughout their medical training. Selecting an acceptable approach for teaching a well-prepared antibiotic stewardship program is critical for successful antibiotic education. This study assesses medical students’ knowledge of appropriate antibiotic prescribing utilizing virtual online antibiotic stewardship education.

## 2. Results

### 2.1. Participants’ Demographic Characteristics

Thirty-three participants were selected to be enrolled in this study. They responded positively to the dispensed survey; 15 (54.1%) were males, while 18 (45.9%) were females. The mean age of the respondents was 22.6 years (SD ± 2.17). The demographic characteristics of the participants are presented in Table 1. Furthermore, students were asked to answer yes or no about their previous experience and education in microbiology, pharmacology, and antibiotic-related courses. No respondents had prior antibiotic stewardship experience, as shown in Table 1.

### 2.2. Medical Students’ Confidence in Antibiotic Prescription and Administration

**Figure 1:**
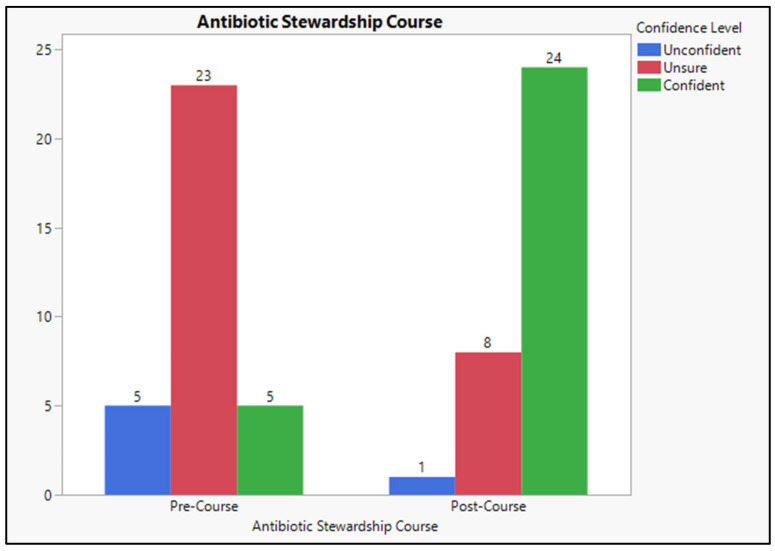
Confidence associated with antibiotics prescription and administration.

Two-thirds of the participants felt unsure and unconfident in prescribing an antibiotic by themselves and accurately diagnosing an infection that needs antibiotic treatment (36.4%) and (33.3%). Moreover, half of the participants (51.5%) failed to understand not prescribing antibiotics for fever without severe criteria and uncertain diagnosis of infection. Interestingly, a high percentage (94%) of the participants thought they were unconfident and not sure if asked to participate in selecting the appropriate antibacterial agents. Moreover, a similar proportion of the participants (94%) felt unconfident in knowing the suitable dosage for antibiotic treatment for a specific indication such as pneumonia or an exacerbation of COPD, and 90% felt uncertain about establishing treatment duration for any antibiotics. Interestingly, a similar percentage (91%) of the participants thought they were unconfident if asked to participate in prescribing combination therapy and establishing treatment duration, as shown in Table 2 and Figure 1.

### 2.3. Medical Students’ Knowledge Associated with the Prescribing and Administering of Antibacterial Agents

#### 2.3.1. Pre-Course Assessment Report

Regarding participants’ knowledge of administering antibiotics and antibiotic resistance, five case scenarios with two to three multiple-choice questions were used to assess the participant’s knowledge. In the first-case scenario, two-thirds (66.7%) of students correctly identified the best and most appropriate treatment recommendation but could not correctly identify the most appropriate treatment duration. For the second scenario, less than half of the participants (48.5%) were able to select the best treatment recommendation, and a similar percentage (45.45%) were able to figure out the recommended duration for the antibiotic. However, only a few (12.1%) could correctly identify the most appropriate treatment duration. Similarly, for the third scenario, only a third of the participants (33.3%) could select the appropriate treatment choice, appropriate administering time, and best treatment recommendation. For the fourth scenario, more than half of the participants (54.6%) could not select the best initial choice of treatment if the goal was to obtain infection rate control or figure out the recommended treatment based on the patient’s history. However, over two-thirds (84.9%) could not correctly select the recommended prophylactic antibiotic. In the last scenario, more than 70% of the participants could not select the appropriate treatment and management, regardless of their detailed history of overseas travel and anticoagulant medications.

#### 2.3.2. Post-Course Assessment Report

**Figure 2:**
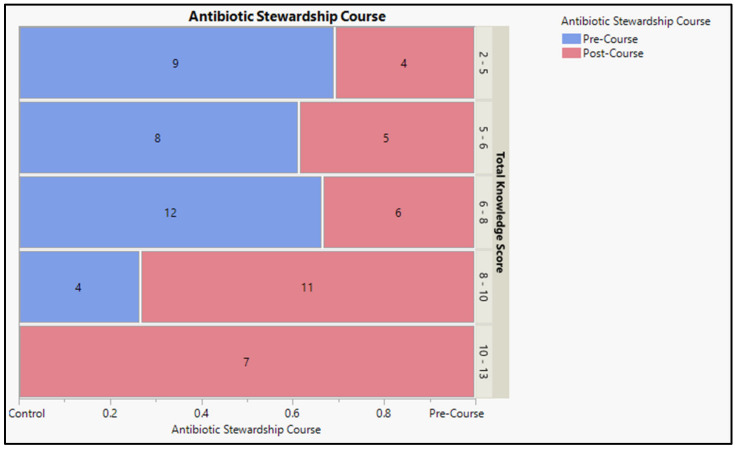
Knowledge associated with antibiotics prescription and administration.

In the first scenario, more than two-thirds (81.8%) of students correctly identified the best and most appropriate treatment recommendation, and (42.4%) correctly identified the most appropriate treatment duration. For the second scenario, more than half of the participants (66.67%) were able to select the appropriate initial course of treatment, and 57.6% were able to figure out the recommended duration for the antibiotic. However, only almost a third (39.4%) could correctly identify the most appropriate treatment duration. For the third scenario, more than half of the participants could select the appropriate treatment choice, administering time, and best treatment recommendation. Similarly, for the fourth scenario, more than half of the participants (54.6%) could select the best initial treatment choice if the goal was to obtain infection rate control and determine the recommended treatment based on the patient’s history. However, only a third (33.3%) could not select the treatment recommendation for the dental appointment. More than half (51.5%) of participants could select the most appropriate treatment for the last scenario. At the same time, almost a third could select the appropriate management based on the detailed history of overseas travel and anticoagulant medications (Table 3 and Figure 2) and detailed pre- and post-course knowledge assessment.

### 2.4. The Level of Familiarity with the “Antibiotic Stewardship” Term

**Figure 3:**
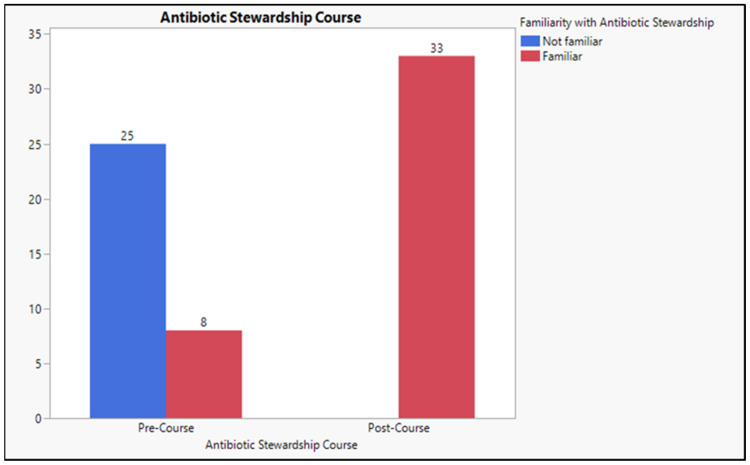
Participants’ familiarity with the antibiotic stewardship term.

Participants were asked about their familiarity with “antibiotic stewardship” in the knowledge section. Almost 70% of participants were unfamiliar with antibiotic stewardship, while 30% had heard about it. Thus, all participants (100%) were introduced to the term in the online courses after the course, as shown in Figure 3.

### 2.5. Association of Confidence, Knowledge before and after the Antibiotic Stewardship Course

The association between confidence and knowledge was measured before and after the antibiotic stewardship course. First, the McNemar–Bowker test analysis revealed a significant difference in students’ confidence before and after enrolling in the online educational program (Z = 20, *p* < 0.002). Second, the matched paired *t*-test was used to compare knowledge scores before and after the course. The table demonstrates a comparison between the mean knowledge scores before and after the courses, and it was found that the mean knowledge score level after the course was higher than the mean knowledge score before the course (M = 7.66 versus M = 5.36 respectively), *t* = 3.540 and *p* < 0.001. Table 4.

## 3. Materials and Methods

### 3.1. Study Design and Setting

**Figure 4:**
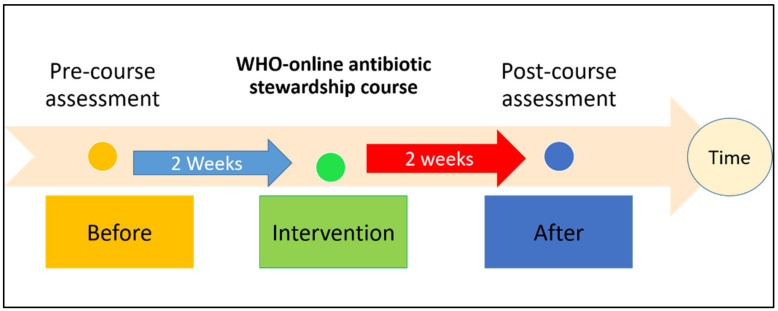
Quasi-experimental one-group pretest-posttest design.

The study design is a quasi-experimental, one-group pretest-posttest design. This design is widely used in clinical, educational, and social research to examine the effect of an intervention. The result of interest is assessed twice: once before and once after exposing a non-random group of participants to a specific intervention [32]. Thus, based on this design, we recruited a group of medical students from the College of Medicine, KSAU-HS, in Jeddah between October and December 2020. KSAU-HS is a public university specializing in health sciences accredited by the Ministry of Education in the Kingdom of Saudi Arabia for its medical program. The group of students was pre-tested utilizing a validated electronic questionnaire adopted from Abbo et al. [30]. Then, the group was enrolled in a WHO-online antibiotic stewardship course. Upon completing the course and providing their certificates as evidence, they were retested using the same instrument. Figure 4 details the experimental process.

### 3.2. Sample Size and Participant Sampling

The projected number of students required was determined using the G*Power statistical software [33]. Therefore, with a 95% confidence interval, +5 standard deviations, 5% difference, and 80% power of the test, the required sample size was determined to be 34 students in one group. Both genders, aged between 20 and 28 years old, were eligible to be selected to participate in this study, and a non-probability convenience sampling method was used for the selection. However, students who had prior exposure to antibiotic stewardship content were excluded.

### 3.3. WHO Antimicrobial Stewardship Course

This online massively open course focuses on antimicrobial stewardship, emphasizing the importance of responsible antibiotic use in healthcare. The course addresses the widespread misuse of antibiotics and its role in the emergence of drug-resistant organisms. It emphasizes the need for healthcare professionals to become stewards of antimicrobials by prescribing them appropriately and educating patients and colleagues. The course consists of 15 modules covering foundational clinical knowledge and practical scenarios for applying antimicrobial stewardship principles. By the end of the 8-hour course, the participants will have a deep understanding of antimicrobial stewardship core competencies and how to apply them in real-world clinical situations.

### 3.4. Data Collection Tools

In this experimental design, the pre-test and post-test were conducted using an electronic questionnaire. It evaluated students’ confidence and knowledge toward appropriate antibiotic prescription and administration; it also measured the impact of the introduced course on their learning outcomes. The data from the students were collected via an online survey using Microsoft Forms. The questionnaire was distributed online in English to all medical students via official e-mail by a research group within the Jeddah campus. The validated electronic questionnaire was adopted from Abbo et al. [30]. It has 111 items and comprises three sections: the first contains questions about demographics, the second contains statements about confidence associated with administering antibiotics, and the third contains knowledge questions.

Confidence-associated questions have a Likert scale with three choices: unconfident, unsure, and confident. The primary outcome was the confidence level, which was taken as a total ranging from 11 to 33. Thus, the overall confidence regarding antibiotic prescription and administration is represented and rated on a three-point categorical scale as unconfident (1.0–1.7), unsure (1.8–2.4), and confident (2.5–3). All participants’ informed consent was duly obtained before participation, and participants were assured that all responses would remain confidential. For students’ knowledge, five case sensations with 15 questions were utilized to evaluate students’ knowledge regarding antibiotic prescribing and administration. The correct selections received one mark, while no points were given to the wrong selections. The overall knowledge score was evaluated as poor <60% or good >60%.

### 3.5. Statistical Analysis

Qualitative data were reported as frequency and percentage, whereas continuous variables were provided as mean, standard deviation (SD), median, minimum, and maximum as appropriate. The McNemar–Bowker Test was performed to compare categorical data and analyze the relationship between confidence levels before and after the course. The matched data paired *t*-test was used to compare knowledge scores before and after the course. The significance level was <0.05. The collected data were tabulated and statistically analyzed using JMP Statistics for Windows, version 15.

## 4. Discussion

Infectious disease emerges and reemerges in the community despite considerable progress and achievements in antibiotic discovery and infection control [6]. Malpractice of antibiotic stewardship by physicians, pharmacists, and consumers is the leading cause of the global increase in antibiotic resistance [4]. Additionally, medical interns lack specific knowledge and skills about the safe practice of antibiotic stewardship; thus, this study aimed to evaluate medical students’ current knowledge and confidence and compare the learning outcomes of the WHO’s antibiotic stewardship course among medical students at KSAU-HS, Jeddah-Saudi Arabia. This study reported significant differences between medical students’ confidence and knowledge toward antibiotic prescription and administering before and after receiving the WHO’s online antibiotic stewardship course.

In line with the recent survey, medical students might experience a shallow confidence level about antibiotic options, the right prescription, and dosing treatment [14]. Our data showed that most participants feel unsure and unconfident about knowing the suitable duration for antibiotic treatment for a specific indication such as pneumonia or exacerbating COPD or establishing treatment duration for any antibiotics. Lower confidence can be attributed to several factors such as lack of proper education; some students may not receive comprehensive education or training on antibiotic use during their medical or healthcare training. This results in uncertainty when prescribing or administering antibiotics. Moreover, some were introduced to antibiotic resistance and might feel fear and pressure to avoid or minimize unnecessary antibiotic prescriptions. In addition, prescribing antibiotics requires critical consideration of patients’ medical history, weight, age, and previous allergies. Thus, prescribing and administering antibiotics might be hard for students who lack clinical experience, proper training and enough exposure.

Researchers have also argued that people are still dying of bacterial infection regardless of the availability of several classes and types of antibiotic agents. Dellinger R.P. et al., 2012, found a high incidence of inappropriate antibiotic management in prescribing among medical practitioners [7]. For the knowledge analysis, the group’s baseline and background knowledge were reported to be lower than what was offered after introducing the virtual course. This was justified by insufficient knowledge and training. Medical and healthcare education programs may not always provide comprehensive antibiotic training, including their appropriate use, indications, dosages, and potential side effects. This can leave students with gaps in their knowledge. Moreover, it might be explained by and refer to the difficulty index of these questions and the complexity of the topic. Understanding the mechanisms of infection, the types of bacteria involved, and the principles of antibiotic resistance can be complex. Some students may struggle to grasp these concepts fully.

Based on the experimental design, students’ confidence and knowledge were assessed before their enrolment in the online educational course about antibiotic stewardship offered by the WHO, while a similar measure was conducted two weeks after completion [28]. Analyzing the students’ confidence before and after completing the online course revealed that the students scored 5 (15.15%) for pre-course and 25.0 (72.73%) for post-course, *p* < 0.001, which is significant. The findings of this study are similar to those of David et al., 2017, which indicated that antibiotic stewardship promotes appropriate antibiotic use to reduce the malpractice of antibiotics [34]. Comparing the students’ knowledge before and after the online course reveals that students scored M = 25.19, SD = 3.54 pre-course and (M = 41.80, SD = 3.54) post-course. There was a significant increase in the students’ knowledge regarding antibiotic prescribing and administering, *p*-value = 0.0004, which is considered significant. Knowledge analysis indicates that the course impacts the students’ knowledge. Thus, offering extensive antibiotic stewardship education might help reduce the number. These results agree with Weier et al., 2017, which examined medical students’ attitudes and understanding of antimicrobial stewardship. It emphasized the need for increased education in this area and the possible influence on prescribing confidence [35]. The term “antibiotic stewardship” was unfamiliar to 70% of the participants, who believed in introducing online courses and integration guidelines in the medical curriculum. There was a significant difference in students’ familiarity with antibiotic stewardship terms before and after enrolling in the online educational program (30.0 versus 100.0, *p* < 0.001 *).

Not many studies in Saudi Arabia have focused on the depth of medical students’ knowledge and confidence in prescribing antibiotics, which this study has done. This study identified the impact of antibiotic stewardship, online courses, and self-education on medical students’ online expertise and enthusiasm and the need to implement effective online educational programs, curricular activities, and awareness campaigns to augment the learning process effectively. Even though this study was conducted based on non-random selection, it is the only experimental design that can establish cause-and-effect relationships between an independent and dependent variable [36]. Several limitations were encountered: first, the course duration was eight hours of online education, and participation was low due to fatigue. Second, although we did our best to distribute the questionnaire among medical students across the college, most respondents were busy; this study coincided with academic midterms, quizzes, and assessments. Third, the risk for maturation could be expected because the academic curriculum ran longitudinally with the course. Similarly, instrumental decay was an essential factor that threatened the internal validity of the questions [37].

Potential future directions for knowledge gaps and lack of confidence: medical programs can provide more comprehensive training on antibiotic use, emphasize the importance of evidence-based practices, and provide opportunities for students to gain clinical experience and build confidence in their decision-making skills. Furthermore, healthcare education programs should prioritize comprehensive antibiotic use training, including regular guidelines and best practice updates. Additionally, ongoing education and awareness campaigns in clinical rotations and practical experiences should allow students to apply their knowledge in real-world scenarios and help them make more informed decisions regarding antibiotic prescription and administration.

## 5. Conclusions

Medical students often lack confidence and understanding when regarding antibiotic selection, prescription, and administration. Their understanding of safe practices within antibiotic stewardship programs is often limited. As a result, it is imperative to take proactive measures to ensure that our future physicians receive a comprehensive education in the principles and practical aspects of judicious antibiotic use. Various potential interventions can be employed to enhance students’ knowledge and confidence. Firstly, it is evident that the existing medical curriculum alone does not adequately equip students with the proficiency to understand, prescribe, and interpret antibiotics upon graduation. Thus, it is strongly recommended to integrate antibiotic-related knowledge into their medical education journey. Secondly, this research underscores the pressing need to develop dedicated educational courses on antibiotic stewardship. These suggested courses could significantly bolster students’ comprehension and confidence in antibiotic prescribing within the context of future patient care. By fostering a deeper appreciation for antibiotics, such initiatives can play a pivotal role in minimizing microbial resistance, improving patient outcomes, and curbing the spread of multidrug-resistant organisms. In summary, enhancing medical students’ proficiency in antibiotic usage and stewardship is a critical endeavor, and it holds the potential to bring about substantial positive impacts in healthcare practice.

## Figures and Tables

**Table 1:** Demographical data of the participants.

Demographic Variables	*N*	(%)
Age (mean + SD) years	22.6 ± 2.17	
Gender		
Male	15	45.5%
Female	18	54.6%

Abbreviations: SD = Standard deviation.

**Table 2:** Confidence associated with antibiotics prescription and administration.

How Confident Do You Feel in the Following Scenarios When Prescribing an Antibiotic by Yourself?	Confidence Level	Antibiotic Stewardship			
		Pre-Course		Post-Course	
		*N*	%	*N*	%
Accurately diagnosing an infection that needs antibacterial treatment	Unconfident	12	36.4	3	9.1
	Unsure	11	33.3	8	24.2
	Confident	10	30.3%	22	66.7
2. Accurately diagnosing community-acquired pneumonia	Unconfident	9	27.3	2	6.1
	Unsure	11	33.3	7	21.2
	Confident	13	39.4	24	72.7
3. Not prescribing antibiotics for fever without severity criteria and uncertain diagnosis of infection	Unconfident	14	42.4	7	21.2
	Unsure	9	27.3	9	27.3
	Confident	10	30.3	17	51.5
4. Selecting the appropriate antimicrobial agents	Unconfident	17	51.5	6	18.2
	Unsure	14	42.4	13	39.4
	Confident	2	6.1	14	42.4
5. Selecting the appropriate route of administration	Unconfident	16	48.5	3	9.1
	Unsure	9	27.3	11	33.3
	Confident	8	24.2	19	57.6
6. Selecting an appropriate dosage regimen for a specific indication, such as pneumonia or chronic obstructive pulmonary disease (COPD) exacerbation	Unconfident	24	72.7	10	30.3
	Unsure	7	21.2	15	45.5
	Confident	2	6.1	8	24.2
7. Knowing the suitable duration for antibiotic treatment for a specific indication such as pneumonia or chronic obstructive pulmonary disease exacerbation	Unconfident	21	63.6	7	21.2
	Unsure	9	27.3	11	33.3
	Confident	3	9.1	15	45.5
8. Prescribing combination therapy	Unconfident	24	72.7	8	24.2
	Unsure	6	18.2	13	39.4
	Confident	3	9.1	12	364
9. Modifying antimicrobial treatment	Unconfident	24	72.7	8	24.2
	Unsure	7	21.2	11	33.3
	Confident	2	6.1	14	42.4
10. Establishing treatment duration	Unconfident	27	81.8	5	15.2
	Unsure	4	12.1	12	36.4
	Confident	2	6.1	16	48.5
11. Identifying situations where antibiotic treatment is not necessary	Unconfident	12	36.4	2	6.1
	Unsure	10	30.3	5	15.2
	Confident	11	33.3	26	78.8
Total Confidence Level	Unconfident	5	15.2	1	3.0
	Unsure	23	69.7	8	24.2
	Confident	5	15.2	24	72.7

**Table 3:** Knowledge associated with the administration of antibiotics and their resistance.

Please Answer the Following Case Scenarios Regarding Antimicrobial Use and Resistance		Score	Antibiotic Stewardship			
			Pre-Course		Post-Course	
			*N*	%	*N*	%
Case 1: Areej, a 39-year-old female with mild COPD, was managed with PRN salbutamol MDI. She has no allergies and is not pregnant or breastfeeding. She presented to her doctor with pain and stinging when urinating and symptoms of urgency. She is diagnosed with an acute, uncomplicated urinary tract infection, and her doctor decides to prescribe her trimethoprim 300 mg daily. Over the next two days, Areej developed a fever and became increasingly unwell. She was diagnosed as having sepsis and was admitted to hospital.	How long should this treatment continue? A. One day B. Three days C. Seven days D. 17 days	Wrong Answer	22	66.7	19	57.6
		Correct Answer	11	33.3	14	42.4
	What would be the most appropriate treatment? A. IV ceftriaxone 2 g daily + IV azithromycin 500 mg daily + IV metronidazole 500 mg twice daily. B. IV benzylpenicillin 1.2 g daily + oral nitrofurantoin 100 mg four times daily C. Oral amoxicillin/clavulanic acid 875 mg/125 mg twice daily + oral doxycycline 100 mg twice daily D. V cefotaxime 2 g three times daily + oral azithromycin 500 mg daily	Wrong Answer	11	33.3	6	18.2
		Correct Answer	22	66.7	27	81.8
Case 2: Sara is a 2-year-old girl with a weight of 12 kg who has presented with ear pain in her left ear, which started the night before. She is healthy despite a history of asthma, can still play and eat, and has no medication allergies. Upon examination, you diagnose her with acute otitis media. Two days later, Sara returns for review. Her ear pain is no better, and she feels tired and irritated as she hasn’t been able to sleep for the past few days.	What is the most appropriate initial course of treatment? A. Amoxycillin 300 mg every eight hours B. Amoxycillin 300 mg + clavulanic acid C. Symptomatic treatment with paracetamol, and advise the parents to return for review if symptoms are still present after two days D. Refer her to a specialist, as it is unusual for children her age to have ear infections	Wrong Answer	17	51.5	11	33.3
		Correct Answer	16	48.5	22	66.7
	How long should antibiotic treatment continue for Sara? A. Amoxycillin 360 mg every six hours B. Amoxycillin 180 mg every eight hours C. Symptomatic treatment with paracetamol for two additional days D. Refer her to a specialist for further assessment	Wrong Answer	18	54.6	14	42.4
		Correct Answer	15	45.5	19	57.6
	How long should antibiotic treatment continue for Sara? A. Two days B. Five days C. Seven days D. Ten days	Wrong Answer	29	87.9	20	60.6
		Correct Answer	4	12.1	13	39.4
Case 3: Ahmad is a 61-year-old male with a history of asthma, COPD, hypertension, hyperlipidemia, and osteoarthritis. He presents to the hospital’s emergency department with breathlessness from any extra exertion over the last three days. He uses his salbutamol inhaler several times each hour and is not getting relief. Ahmad had increasing sputum purulence, and antibiotics were prescribed. While Ahmad was in the hospital, there was a worldwide shortage of rosuvastatin, and the hospital could not source it anywhere. A decision is made to prescribe simvastatin instead. After one week, Ahmad is feeling much better, and a decision is made to discharge him.	What would be the treatment of choice for Ahmad? A. Amoxycillin/clavulanic acid 875 mg/125 mg 1 tablet bd for five days B. Azithromycin 500 mg daily for three days C. Amoxycillin 500 mg tds for five days D. Cefuroxime 500 mg bd for five days	Wrong Answer	22	66.7	22	66.7
		Correct Answer	11	33.3	11	33.3
	At what time of day will it have its maximum efficacy? A. In the evening B. In the morning C. It should be taken twice a day D. It doesn’t matter what time of day it is taken—it will still have the same efficacy	Wrong Answer	22	66.7	15	45.5
		Correct Answer	11	33.3	18	54.6
	What would not be a recommended medication for Ahmad? A. Cephalexin 250 mg daily ongoing B. Salbutamol 100 mcg MDI 1–2 puffs prn C. Tiotropium 18 mcg one capsule inhaled daily D. Fluticasone/Salmeterol 125 mcg/25 mcg MDI 2 puffs bd	Wrong Answer	21	63.6	16	48.5
		Correct Answer	12	36.4	17	51.5
Case 4: Khalid is a 25-year-old male of nomadic origin. He lives a basic lifestyle, including cooking over campfires, bathing in the nearby river, and sleeping in makeshift tents. Several months ago, he had recovered from a high fever and had pain in multiple joints. He has presented again with the same symptoms, experiencing ongoing fatigue and dyspnea. He was diagnosed with rheumatic heart disease and developed atrial fibrillation. Several years have passed, Khalid is managing well. However, he had recently been to the dentist for a tooth extraction.	What would be the initial treatment choice if the goal is to obtain rate control? A. Metoprolol 25 mg bd B. Verapamil SR 160 mg daily C. Diltiazem SR 360 mg daily D. Amiodarone 200 mg tds	Wrong Answer	18	54.5	12	36.4
		Correct Answer	15	45.5	21	63.6
	What would be the recommended antibiotic prophylactic? A. Amoxycillin 2 g orally 1 h before the procedure B. Amoxycillin 2 g orally 1 h before the procedure, then 500 mg tds for five days C. Amoxycillin 500 mg tds for five days following the procedure D. The patient doesn’t require prophylactic antibiotics in this case	Wrong Answer	28	84.8	17	51.5
		Correct Answer	5	15.2	16	48.5
	What would your recommendation be if Khalid reported that he experienced an urticaria and bronchospasm reaction to benzylpenicillin in the past? A. Amoxycillin 2 g orally 1 h before the procedure B. Cephalexin 2 g orally 1 h before the procedure C. Clindamycin 600 mg orally 1 h before the procedure D. The patient should not be given prophylactic antibiotics in this case	Wrong Answer	18	54.5	11	33.3
		Correct Answer	15	45.5	22	66.7
	What would your treatment recommendation be if Khalid was having dental impressions and the construction of dentures done? A. No prophylactic antibiotics are needed B. Amoxycillin 2 g orally 1 h before the procedure C. Amoxycillin 2 g orally 1 h before the procedure, then 500 mg tds for five days D. Amoxycillin 500 mg tds for five days following the procedure	Wrong Answer	19	57.6	22	66.7
		Correct Answer	14	42.4	12	33.3
Case 5: Mona is a 55-year-old woman complaining of diarrhea for the past three days. She had recently returned home from Vietnam, and since then, she has been feeling nauseous and feverish for several days before the diarrhea. She takes metformin 500 mg daily for type II diabetes and warfarin at night for deep vein thrombosis.	What would be the most appropriate management of Mona? A. Azithromycin 500 mg orally daily for three days B. Ciprofloxacin 500 mg orally bd for three days C. Amoxycillin/Clavulanic acid 875 mg/125 mg orally bd for five days D. Antibiotic treatment is not indicated	Wrong Answer	25	75.8	16	48.5
		Correct Answer	8	24.2	17	51.5
	What would your treatment recommendation be if Margaret had not reported any recent overseas travel? A. Azithromycin 500 mg orally daily for three days B. Amoxicillin/Clavulanic acid 875 mg/125 mg bd orally for five days C. Ciprofloxacin 500 mg orally bd for three days D. Antibiotic treatment is not indicated	Wrong Answer	24	72.7	22	66.7
		Correct Answer	9	27.3	11	33.3
	Mona is also taking warfarin; what other advice would you give her at this stage? A. As she is currently unwell, she should stop taking warfarin and only recommence it once she is feeling better B. She should not take her warfarin at the same time as taking antibiotics as if she gets nauseous with the antibiotic, and the warfarin won’t be absorbed as well C. All antibiotics can decrease the INR, so the warfarin dose should be doubled during treatment D. She will require regular monitoring of her INR, as being unwell may affect her INR result, and she may require dosage adjustments during this time	Wrong Answer	24	72.7	20	60.6
		Correct Answer	9	27.3	13	39.4

Abbreviations: COPD: Chronic obstructive pulmonary disease, TDS: 3 times a day, bd: twice per day.

**Table 4:** Association of confidence and knowledge before and after the antibiotic stewardship course.

		Pre/Post	Unconfident	Unsure	Confident	Test Value	*p* Value
Total Confidence Level	Confidence Level	Unconfident	0 (0.00%)	3 (9.09%)	2 (6.06%)	Symmetry disagreement *Z* = 20	0.002 *
		Unsure	1 (3.03%)	5 (15.15%)	17 (51.52%)		
		Confident	0 (0.00%)	0 (0.00%)	5 (15.15%)		
Total knowledge score			Mean score	Pre-Course	Post-Course	*t* = 3.540	<0.001 **
				5.36 ± 1.74	7.66 ± 2.59		

* McNemar–Bowker test and ** Matched Paired *t*-test.

## Data Availability

The data used to support the findings of this study are restricted by the Institutional Review Board (IRB) and the Research Board of King Abdullah International Medical Research Center (KAIMRC) of King Abdullah International Medical Research Center (KAIMRC) IRBC/1951/20. The datasets generated during and analyzed during the current study are available from the corresponding author for researchers upon reasonable request.
